# Beyond monotherapy: Combination therapies for HIV-1 cure through joint application of neutralizing antibodies, genome editing, and reservoir management

**DOI:** 10.1016/j.imj.2025.100215

**Published:** 2025-11-05

**Authors:** Qiongbin Mao, Lin Li, Hongling Wen

**Affiliations:** a Department of Microbiological Laboratory Technology, School of Public Health, Cheeloo College of Medicine, Shandong University, Key Laboratory for the Prevention and Control of Emerging Infectious Diseases and Biosafety, Jinan 250012, Shandong Province, China; b State Key Laboratory of Pathogen and Biosecurity, Academy of Military Medical Sciences, Beijing 100071, China

**Keywords:** Envelope trimer vaccine, Multimodal synergistic strategy, HIV-1 cure, Broadly neutralizing antibody, Latent HIV-1 reservoir

## Abstract

Despite its potency in suppressing HIV-1 replication, antiretroviral therapy (ART) cannot eliminate latent viral reservoirs and is associated with several limitations, such as the need for lifelong treatment and the inherent risk of drug resistance. The quest for an HIV-1 cure has progressed from monotherapeutic approaches to the combinations of multimodal strategies, including neutralizing antibodies, precision genome editing, and management of latent reservoirs. Antibody-based interventions primarily involve inducing broadly neutralizing antibodies (bNAbs) through native-like envelope (Env) trimer vaccines, with their efficacy further enhanced by mRNA-lipid nanoparticle delivery systems. Precision genome editing can be achieved by using clustered regularly interspaced short palindromic repeats (CRISPR)/CRISPR-associated protein (Cas) along with long-acting slow-effective release antiretroviral therapy. Reservoir-targeted therapies are typically implemented by reactivating latent viruses using the “shock and kill” strategy. Engineered cellular therapies include chimeric antigen receptor T (CAR-T) cells or bispecific antibodies (bsAbs) for subsequent immune clearance, immune system reconstitution via stem cell transplantation, and reversal of T-cell exhaustion using immune checkpoint inhibitors. Despite these advances, challenges remain, including suboptimal immunogenicity of Env vaccines, off-target effects and inefficient delivery of gene editing tools, incomplete reactivation of latent viruses, and limitations of preclinical models. Future research should focus on optimizing synergistic effects by improving Env trimer design, enhancing the targeting specificity of CRISPR systems, and developing preclinical models that more accurately reflect human immunity, thereby facilitating the transition from lifelong ART to a functional cure. This review summarizes recent progress in multimodal synergistic strategies and proposes a framework for an HIV-1 cure that may also offer insights into the treatment of other chronic viral infections.

## Highlights

• This review proposes a novel combinatorial strategy for HIV-1 cure.
• The mRNA-LNP vaccine induces broadly neutralizing antibody precursors for broad-spectrum suppression.
• The CRISPR-Cas9 synergizes with long-acting ART to excise the HIV reservoir.
• The combination of engineered CAR-T cells and LRAs achieves significant viral load reduction.
• A three-level cure system guides future HIV treatment development.

## Graphical abstract

**Figure:**
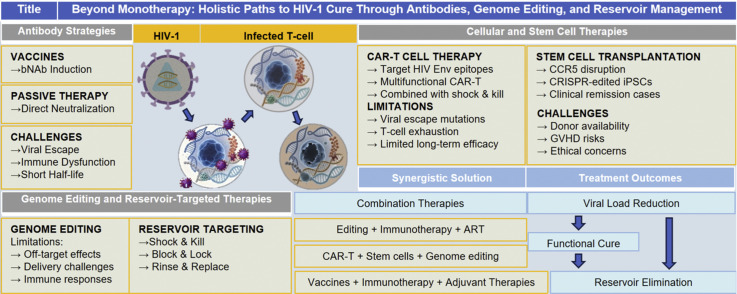

## 1 Introduction

The feasibility of curing HIV-1 infection was first demonstrated in the Berlin and London patients, who achieved sustained viral remission through C—C chemokine receptor type 5 (CCR5)-Δ32 homozygous hematopoietic stem cell transplantation (HSCT) while being treated for hematologic malignancies. However, this HSCT-based strategy has limited generalizability, mainly due to the rarity of CCR5-Δ32 homozygous donors and the high risks associated with allogeneic transplantation.^1^ Although a small subset of “elite controllers” can maintain viral suppression without ART, the vast majority of people living with HIV-1 (PLWH) remain reliant on lifelong ART. Even with ART, they face persistent challenges, such as low-level viremia and rapid viral rebound following ART discontinuation.^2^

As of 2023, approximately 39.9 million PLWH exist worldwide, among whom 30.7 million are receiving ART. HIV-1 treatment regimens have evolved from daily oral tenofovir disoproxil fumarate/emtricitabine (TDF/FTC) to the annually administered capsid inhibitor lenacapavir, marking a transition from daily oral formulations to long-acting agents. This progress indicates that HIV-1 treatment has entered the “post-ART era”—a phase focused on moving beyond lifelong daily oral ART toward cure or long-term remission strategies.^3^ Consequently, research focus has shifted towards multimodal therapeutic strategies, which integrate three core pillars: antibody-based immune modulation, precision genome editing, and latent HIV-1 reservoir management. Based on clinical feasibility and outcomes, HIV-1 treatment goals are categorized into three tiers: functional cure (sustained ART-free control of viremia and disease progression despite persistent proviruses), hybrid cure (partial reservoir clearance combined with immune regulation, requiring long-term monitoring), and sterilizing cure (complete elimination of replication-competent proviruses).^4^ Future success in HIV-1 cure research will depend on the integration of these technologies.

## 2 Antibody-based strategies: Vaccines and passive immunotherapy

### 2.1 Envelope (Env) trimer vaccines: Design and optimization

#### 2.1.1 Conformational stabilization strategies

The high mutation rate and predominantly closed, dynamic conformation of the HIV-1 Env glycoprotein, which is a critical target for broadly neutralizing antibodies (bNAbs) induction, pose significant challenges for vaccine development.^5^ To overcome these challenges, native-like Env trimer vaccines have been engineered using multiple strategies to enhance bNAbs induction and neutralizing activity. These strategies include modifying gp120 glycosylation sites, targeting conserved regions (e.g., the CD4 binding site), and modulating B-cell maturation pathways.^6^

Recent progress in HIV-1 vaccine design have focused on optimizing the conformations of Env trimer to elicit potent bNAbs. Initial vaccine candidates failed due to non-native gp41 structures, which led to the development of soluble, cleavage-independent, prefusion-optimized (SOSIP)-stabilized trimers. These trimers incorporate proline mutations at critical gp120-gp41 interfaces to stabilize the native conformation. The BG505 SOSIP trimer is a prime example of this progress, exhibiting broad bNAbs recognition while minimizing the induction of non-neutralizing antibodies that target the membrane-proximal external region.^7^ Subsequent refinements, such as the SOSIP.664 and SOSIP.v9 variants, have achieved improved thermal stability and reduced binding of non-neutralizing antibodies, thereby eliciting Tier-2 neutralizing responses in animal models.^8^

To address persistent challenges, novel stabilization strategies have been developed. Interdomain-locked trimers maintain native-like conformations through intramolecular disulfide bonds, although this approach results in impaired CD4 binding. In contrast, naturally flexible linkage trimers preserve the authentic dynamics of the Env protein by optimizing gp120-gp41 linkages.^9^^,^^10^ Complementing these advances, supramolecular nanofibers improve immunogenicity by precisely organizing gp120 subunits in space.^11^

#### 2.1.2 Delivery system innovations

These structural innovations are being combined with optimized immunization protocols, such as the N332-GT5 prime followed by B11/B16 boost, which effectively elicit germinal center (GC) responses in non-human primates.^12^ While traditional aluminum-adjuvanted subunit vaccines are limited by non-native epitope presentation, next-generation delivery systems demonstrate significant potential. For instance, polymeric nanoparticles, such as the eOD-GT8 60-mer combined with the AS01B adjuvant, mimic the natural distribution of antigens;^13^ virus-like vaccines combine the benefits of virus-like particles and viral vectors, enabling sustained antigen expression.^14^ Notably, mRNA vaccines encapsulated in LNPs enable direct cytoplasmic antigen translation, efficiently recruiting B cells and inducing high-affinity neutralizing antibodies. Phase I trials (IAVI-G002/003) have confirmed their capacity to safely promote the maturation of bNAbs precursors in humans.^15^ Collectively, these advanced delivery platforms offer a multifaceted approach to overcoming longstanding barriers in HIV-1 vaccine development.

### 2.2 Passive immunotherapy: Broadly neutralizing antibodies

#### 2.2.1 Clinical efficacy and limitations

Although active immunization via vaccines is the ideal approach for long-term HIV control, it faces significant challenges. Consequently, passive immunotherapy with bNAbs remains a valuable clinical tool, despite its inherent limitations. bNAbs have demonstrated efficacy: infusion of 3BNC117 improved neutralizing responses to heterologous Tier 2 viruses in nearly all participants, enhancing host humoral immunity; combinations of two bNAbs have also temporarily controlled viremia in rhesus macaques;^16^ and a three-bNAb regimen reduced viremia in ART-naive individuals in a Phase I trial (NCT03205917).^17^ However, the effects of bNAbs are transient due to viral escape, persistent reservoirs, and their short half-lives. Therefore, passive immunotherapy with bNAbs always need frequent (often lifelong) injections. These issues have only been partially addressed by half-life-extending modifications or bispecific engineering. These limitations underscore that neither active immunization alone nor passive bNAb therapy is sufficient. Instead, combination with ART is critical for durable HIV-1 control.^18^

#### 2.2.2 Development of bispecific antibodies

Given these limitations, combination strategies have emerged as a major research focus. bsAbs that target Env protein on infected cells and CD16 on natural killer (NK) cells, have been developed to eliminate residual infected cells. Combining active vaccination (e.g., Env vaccines) with passive immunotherapy (e.g., bNAbs) constitutes a comprehensive “prevention-treatment-clearance” strategy.^19^ However, challenges persist regarding neutralization breadth, bNAb maturation efficiency, and the mechanisms of viral escape from bNAbs, all of which require further investigation.^20^

### 2.3 Translational challenges of antibody-based strategies

Both active immunization via Env vaccines and passive immunotherapy (e.g., with bNAbs and bsAbs) face substantial shared translational hurdles in clinical translation. For Env vaccines, although preclinical studies have shown promising efficacy, their clinical application is impeded due to immune evasion. Specifically, HIV-1 can evade neutralization by accumulating mutations in Env epitopes, which impairs neutralizing activity of both vaccine-induced bNAbs and passively administered antibodies. Another critical barrier is the dysfunction of germinal center T follicular helper (GC-Tfh) cells: HIV-induced impairment of these cells disrupts CD40L-dependent B-cell maturation, which not only limits the production and durability of vaccine-induced bNAbs but also impairs the establishment of immune memory in passive immunotherapies.^21^

These challenges are further exacerbated by limitations in preclinical models, which hinder accurate efficacy prediction for both strategies. Nonhuman primates partially recapitulate HIV-1 pathogenesis but exhibit immune variability that poorly reflects human germinal center responses, whereas humanized mouse models fail to recapitulate long-term viral reservoir dynamics, a critical factor for evaluating both vaccine-induced reservoir control and antibody-mediated clearance.^22^ To address these gaps, researchers are optimizing immunization strategies: For example, a two-dose saponin-adjuvanted Env trimer regimen has elicited bNAbs in mice, and a prime-boost protocol in nonhuman primates using glycan-targeted SOSIP immunogens has improved antigen-specific responses. These advances underscore the necessity of employing optimized models and combinatorial approaches to overcome the shared translational barriers of Env vaccines and passive immunotherapies.^23^^,^^24^

### 2.4 Synergistic strategies: Combining antibodies with other modalities

Given these widespread translational challenges, monotherapeutic antibody-based approaches are unlikely to achieve durable viral suppression. To overcome the limitations of viral escape and a heterogeneous latent reservoir, the field is shifting toward multimodal strategies designed to target multiple vulnerabilities simultaneously. This paradigm shift is the core analytical framework of this review and is implemented through several key mechanisms: First, one key approach involves combining antibodies with reservoir-targeting agents, such as latency-reversing agents (LRAs) or immune checkpoint inhibitors, to enhance the clearance of reactivated HIV-1 reservoirs. Second, the strategy encompasses engineering adoptive cellular therapies like chimeric antigen receptor T (CAR-T) cells to secrete bsAbs, thereby establishing sustained antiviral effector functions. Third, a combined approach coordinates active vaccination, which elicits durable de novo immunity, with passive antibody transfer to provide immediate viral suppression. Fourth, a synergistic strategy integrates antibody-mediated surveillance for active infection with clustered regularly interspaced short palindromic repeats (CRISPR)/CRISPR-associated protein (Cas) 9-mediated proviral excision, directly targeting the genetic basis of the latent reservoir to reduce the risk of viral rebound.

## 3 Genome editing strategies for HIV-1 proviral targeting

### 3.1 CRISPR/Cas9-mediated proviral DNA editing

Gene editing technologies utilize nucleases, including zinc finger nucleases (ZFNs), meganucleases, transcription activator-like effector nucleases (TALENs), and the CRISPR/Cas9 system—to generate site-specific DNA double-strand breaks. These breaks activate cellular repair mechanisms to facilitate targeted genetic modifications. Among these tools, ZFNs, meganucleases, and TALENs rely on protein-DNA interactions for target recognition, as exemplified by ZFN-mediated CCR5 disruption in CD4^+^ T cells.^25^ In contrast, CRISPR/Cas9 offers programmable precision but faces challenges such as off-target effects due to short guide RNA mismatches. Innovations such as nanoparticle-based delivery systems are designed to mitigate these risks.^26^

### 3.2 Delivery systems and clinical translation

Transitioning from mechanism to application, CRISPR/Cas9 paired with dual guide RNAs (gRNAs) has shown efficacy in excising viral DNA in preclinical mouse and macaque models.^27^ For instance, the EBT-101 trial (NCT05144386) assessed this approach in ART-treated PLWH, targeting conserved HIV-1 genomic regions. Phase 1/2 results indicated post-treatment viral rebound, but the trial highlighted the potential of CRISPR for reservoir reduction.^28^ Notably, this rebound clearly underscores two intractable clinical hurdles: the immunogenicity of delivery platforms and the formidable barrier of achieving complete reservoir targeting *in vivo*.

Despite promising preclinical data and initial clinical validation of the reservoir-reducing capacity of CRISPR, even high-fidelity Cas variants and optimized gRNAs (designed to minimize off-target risks) fail to achieve complete HIV eradication. Eliminating HIV entirely requires the delivery of gene-editing machinery to nearly all latently infected cells, a capability that remains beyond current technical reach. Another key barrier is the immunogenicity of delivery vectors, including adeno-associated virus and LNPs, as well as bacterial-derived Cas9. Immune responses can clear edited cells or block re-dosing, thereby compromising long-term efficacy. These limitations underscore the need for ongoing advancements in CRISPR-based strategies. One such advancement is combining CRISPR/Cas9 with LASER ART, which achieved excision of HIV-1 proviral DNA in 58% of tissues in humanized mice, although complete HIV eradication remains elusive.^29^

### 3.3 Combinatorial strategies to enhance efficacy

To enhance the clinical utility of CRISPR, addressing inefficient delivery and immunogenicity is essential. This necessitates a shift from monotherapeutic gene editing to combinatorial strategies that combine CRISPR with immune-based approaches, such as immune checkpoint modulation, to eliminate residual unedited reservoirs. Additionally, optimizing delivery vectors (e.g., LNPs) will be critical to enhance both specificity and clinical efficacy.

Consequently, the path forward for CRISPR-based cure strategies lies not in achieving perfect monotherapeutic gene editing, but in the rational integration of CRISPR with other modalities that offset its inherent limitations. The well-documented challenges of inefficient delivery and latent reservoir heterogeneity provide a compelling rationale for combining CRISPR with immune-based approaches. For instance, the residual population of latently infected cells that inevitably escapes genome editing due to insufficient delivery presents a critical vulnerability: these cells often remain capable of viral antigen expression, making them prime targets for immunotherapy. Thus, a logical synergy exists wherein CRISPR acts as a precise tool for proviral excision within reachable cells, and immunotherapies (e.g., bNAbs, bsAbs, or engineered CAR-T cells) act as a systemic surveillance mechanism to eliminate CRISPR-escaping, antigen-positive cells. Future research must therefore focus not only on improving editing efficiency but also on determining the optimal timing, sequencing, and delivery platforms for these combinations to enable a concerted attack on the latent reservoir.

## 4 Latent HIV-1 reservoir intervention: From single strategies to synergism

### 4.1 Proviral targeting paradigms

HIV-1 latency remains the primary barrier to HIV-1 cure. The “shock and kill” strategy utilizes LRAs to reactivate latent HIV-1, facilitating viral clearance via cytotoxic T lymphocyte responses or antibody-dependent cellular cytotoxicity. Notably, the co-encapsulation of LRAs and bNAbs in polymeric nanoparticles, when combined with NK cells effectively eliminate HIV-infected cells *in vitro*. However, due to impaired immune function in ART-treated individuals, LRAs often exhibit suboptimal efficacy in reactivating latent viruses.^30^ To address this limitation, optimizing combinations of LRAs using CRISPR technology and *in vitro* models, alongside enhancing the “kill” phase with bNAbs, represents a promising direction.^31^ In clinical trials, the LRA chidamide successfully reactivated latent HIV-1 but failed to reduce viremia or the HIV-1 reservoir size when combined with ART.^32^ These repeated failures reveal a fundamental insight: the obstacle is not “shocking” the virus, but rather the immune system's inability to “kill” reactivated HIV-1.

Alternative strategies target the HIV-1 latent reservoir through distinct mechanisms. The “rinse and replace” strategy aims to indirectly reduce the latent HIV-1 reservoir by accelerating cellular turnover. Repeated administration of a toll-like receptor 7 (TLR-7) agonist in simian immunodeficiency virus (SIV)-infected cynomolgus macaques has been shown to reduce the SIV reservoir.^33^ Additionally, DNA vaccines combined with TLR agonists can induce protective T-cell immunity in rhesus macaques, while multivalent strategies further enhance immune responses.^34^ Conversely, the “block and lock” strategy utilizes latency-promoting agents to permanently silence HIV-1 proviruses. Latency-promoting agents, such as LEDGINs (lentiviral early transcriptional inhibitors), inhibit the interaction between viral integrase and the host cofactor LEDGF/p75, thereby preventing viral reactivation.^35^ These strategies highlight the need for combinatorial approaches to overcome the limitations of monotherapy and achieve sustained control of HIV-1 reservoir.

The consistent lack of efficacy of LRA monotherapy in clinical trials underscores a critical insight: the primary barrier to a cure is not reactivating the virus, but rather the functional incompetence of the immune system in ART-treated individuals to clear the infected cells. This redefines the challenge from a virological issue to an immunological one. Consequently, the future of the “shock and kill” strategy must evolve into a multi-step “prime, shock, and kill” strategy. This approach necessitates initial immune reconstitution, for instance, using IL-15 superagonists to expand NK cell populations or immune checkpoint inhibitors to reverse T-cell exhaustion, to establish a potent immune effector response. The subsequent “kill” phase then requires precision targeting with agents such as bsAbs to direct these pre-activated immune effectors toward the reactivated cells. This sequential strategy addresses the root cause of failure by ensuring a functional immune system is in place to eliminate all reactivated HIV-1-infected cells.

### 4.2 Engineered cell therapies for reservoir clearance

Engineered anti-HIV-1 CAR-T cells specifically target epitopes of the HIV-1 Env glycoprotein, thereby enhancing antiviral immune responses.^36^ For example, trifunctional M10 CAR-T cells, which express bispecific CARs and secrete bNAbs, achieved a 67.1% reduction in viral load when combined with a chidamide-based “shock and kill” strategy.^37^ However, despite this potential, anti-HIV-1 CAR-T therapy confronts major clinical hurdles. Early-phase clinical trials show limited efficacy in preventing viral rebound after ART interruption, mainly due to poor *in vivo* persistence and functional activity of CAR-T cells. A key hurdle is the variability of the gp120 target, which leads to viral escape. Other obstacles include insufficient exposure of conserved epitopes on latently infected cells and an immunosuppressive microenvironment that promotes T-cell exhaustion, a key mechanism limiting the sustained efficacy of CAR-T cells in both humanized mouse and nonhuman primate models. Furthermore, the high sequence homology between lentiviral vectors and HIV-1 itself causes significant quantification challenges, which complicates the distinction between true viral replication and vector-derived signals in molecular assays. This interference underscores the urgent need to standardize methods for detecting HIV-1 nucleic acids in the context of lentiviral vector-based therapies.

These issues collectively limit long-term efficacy of CAR-T cell therapy, necessitating a broader recognition scope for CARs and precise targeting of the HIV-1 latent reservoir. A further critical issue exacerbating these challenges is the inherent susceptibility of infused CAR-T cells to HIV-1 infection. This creates a critical paradox: the therapy itself is vulnerable to the virus it is designed to combat, potentially generating new reservoirs.^38^ Thus, the major barriers to anti-HIV-1 CAR-T therapy include viral escape, T-cell exhaustion, and intrinsic cellular susceptibility.

To address viral escape and infection susceptibility, engineering HIV-resistant CAR-T cells represents a promising strategy. For instance, researchers have utilized CRISPR/Cas9 for targeted CAR integration into the CCR5 gene, with adeno-associated virus serotype 6 as the donor vector to improve homology-directed repair efficiency.^39^ In a humanized bone marrow-liver-thymus mouse model, these CCR5-edited dual CD4-CAR-T cells showed superior antiviral activity and durability compared to conventional anti-HIV-1 CAR-T cells.^40^

Combating the immunosuppressive microenvironment and T-cell exhaustion remains a critical focus, as HIV-induced T-cell exhaustion further impairs the viral clearance. Immune checkpoint inhibitors targeting programmed cell death protein 1 **(**PD-1) and cytotoxic T-lymphocyte-associated protein 4 can reverse exhaustion and restore antiviral function, but their effects in ART-treated rhesus macaques were transient, and their clinical efficacy as a standalone cure strategy is limited. To address these limitations, interventions extend beyond pharmacology to innovative cellular engineering, which can directly counter T-cell exhaustion. For instance, engineering CAR-T cells to express PD-1 dominant negative receptors (DNRs) blocks PD-L1 signalling and enhances the killing of HIV-infected cells in preclinical models.^41^ Notably, low-dose intermittent rapamycin (a mammalian target of rapamycin inhibitor) offers a potentially safer alternative strategy. In HIV-infected humanized mice engrafted with CAR-engineered hematopoietic stem cells, rapamycin remodelled the CAR-T cell transcriptome by enhancing mitochondrial function and cytotoxic activity while downregulating immune checkpoint molecules. This reduced chronic inflammation and T-cell exhaustion, improved HIV-1 control, delayed ART rebound, and reduced the size of the HIV-1 latent reservoir.^42^ Adoptive cellular strategies also extend beyond CAR-T therapy; T-cell receptor gene therapy and *in vitro* T-cell expansion are promising alternative approaches, offering distinct mechanisms to target HIV-infected cells.^43^

Beyond overcoming individual limitations, a more integrated synergistic strategy involves engineering CAR-T cells with autonomous multifunctional capabilities. For instance, arming anti-HIV-1 CAR-T cells to secrete HIV-1-specific bNAbs simultaneously addresses the challenges of viral escape and the immunosuppressive microenvironment. The secreted bNAbs can neutralize free HIV-1 virions, thereby preventing viral escape from CAR recognition, and can also opsonize nearby HIV-1 infected cells. This opsonization engages innate immune effectors such as NK cells via antibody-dependent cellular cytotoxicity to eliminate antigen-positive cells residing in immunosuppressive environments that are poorly accessed by CAR-T cells. This “micro-pharmacy” strategy effectively transforms CAR-T cells from solitary hunters into orchestrators of a broader, cooperative immune attack, representing a convergence of cellular and humoral immunity that directly addresses HIV-1 latent reservoir heterogeneity.

### 4.3 Immune reconstitution via hematopoietic stem cell transplantation

Cellular and hematopoietic stem cell (HSC) therapies represent pivotal components of multimodal HIV-1 eradication strategies, synergistically integrating antibody-based interventions, genome editing, and latent reservoir-targeted approaches. These therapies enhance antiviral efficacy via synergistic mechanisms, including CAR-T cells secreting bNAbs, CRISPR-modified stem cells, and combinations with “shock and kill” strategy.

HSCT strategies target HIV-1 resistance via CCR5 disruption, supported by both clinical cases and preclinical research—yet critical limitations compromise their scalability and ethical feasibility. Furthermore, HSCT's high morbidity, mortality, and reliance on rare donors confine it to a last-resort therapy for terminal malignancies, rather than a scalable cure for HIV-1. Clinically, the “Berlin Patient” and “London Patient” achieved long-term HIV-1 remission via CCR5-Δ32 homozygous HSCT,^44^ while the “City of Hope Patient” demonstrated sustained remission with wild-type CCR5 donor cells, uncovering alternative HIV-1 control mechanisms.^45^ Preclinically, CRISPR/Cas9-mediated CCR5 knockout in induced pluripotent stem cells (iPSCs) has validated this strategy by blocking CCR5-tropic HIV-1 infection.^46^

However, critical translational barriers severely limit the scalability and ethical feasibility of this HSCT strategy. The reliance on rare CCR5-Δ32 homozygous donors raises substantial ethical concerns regarding equitable access to treatment and exacerbates health disparities. Furthermore, allogeneic HSCT carries significant risks, including graft-versus-host disease (GVHD), opportunistic infections, and transplant-related mortality, which currently preclude its application beyond patients requiring HSCT for life-threatening hematological malignancies. Additionally, the potential for unintended germline modifications in CRISPR-based strategies—though autologous settings may mitigate this risk—warrants careful ethical scrutiny and robust regulatory oversight. These limitations underscore that hematopoietic stem cell transplantation, in its current form, remains a proof-of-concept rather than a scalable HIV-1 cure, redirecting research focus toward safer, more equitable alternatives such as autologous gene-edited cell therapies.

## 5 Comprehensive analysis and integration of HIV-1 cure strategies

As delineated earlier, current HIV-1 cure strategies can be categorized into three interconnected categories: immune modulation aimed at enhancing antiviral immunity and suppressing viremia; reservoir-targeted interventions to eliminate or silence latent infected cells; and cell replacement/editing to reconstruct HIV-resistant immune systems. Critically, sustained viral remission is not contingent on optimizing a single category, but rather on integration of these approaches—combining direct reservoir targeting, potent immune effectors, and immune reconstitution—to address key monotherapy hurdles: reservoir persistence, viral diversity, and T-cell exhaustion.

Table 1 below contrasts these three core strategy categories across five dimensions: core mechanisms of action, target cure tiers, translational challenges (e.g., HSCT donor scarcity, inefficient CRISPR delivery), preclinical/clinical evidence, and potential synergistic combinations (e.g., bNAbs + CRISPR). It highlights trade-offs between therapeutic objectives and clinical feasibility and identifies complementary combinations to address monotherapy limitations.

**Table 1:** Comparative overview of core HIV-1 cure strategies: Mechanisms, challenges, evidence, and potential synergies.

Strategy category	Representative strategy	Core mechanisms of action	Target cure tier	Key translational challenges	Preclinical/clinical evidence	Potential synergies
Immune modulation	Env trimer vaccines	Induction of bNAbs targeting conserved epitopes	Functional cure	Limited antibody breadth; viral escape mutations; immune dysfunction^21^	BG505 SOSIP elicits tier-2 neutralizing antibodies (NAbs) in animal models^8^	Enhanced immunogenicity in combination with mRNA-LNP delivery system^15^
Immune modulation	Passive bNAb Immunotherapy	Direct viral neutralization	Functional cure	Short half-life; viral escape; requires frequent dosing	Triple bNAb regimen transiently suppresses viremia in ART-naïve individuals (NCT03205917)^17^	Combination with intermittent ART, CAR-T therapy, or active immunization
Immune modulation	Immune checkpoint modulation	Blockade of PD-1/CTLA-4 signaling and reversal of T cell exhaustion	Functional cure	Transient efficacy; potential for immune-related adverse events	ART combined with PD-1 DNR CAR-T clears latent cells in preclinical models^41^	Combination with LRAs or vaccines to enhance killing
Partial reservoir reduction	CRISPR/Cas9	Proviral DNA excision	Hybrid cure	Off-target effects; inefficient *in vivo* delivery; immunogenicity of delivery vectors	Cleared 58% proviral DNA in tissues with LASER ART in humanized mice;^29^ viral rebound observed in the EBT-101 trial (Phase I/II, NCT05144386)^28^	Combination with CCR5-edited stem cell transplantation may enable a sterilizing cure
Partial reservoir reduction	“Shock and Kill” + ART	Latency reversal using LRAs + Immune clearance	Hybrid cure	Incomplete latency reversal; impaired immune effector function	NP-co-delivered LRA/bNAb enhances killing of latent cells *in vitro*;^31^ Chidamide + ART fails reservoir reduction in Phase II trial (NCT03803605)^32^	LRA combined with immune checkpoint inhibition (e.g., using PD-1 DNR CAR-T cells)^41^
Partial reservoir reduction	“Rinse and Replace” + ART	Accelerated cell turnover	Hybrid cure	Potential for immune activation-related toxicity; efficacy in humans unproven	Reduction of SIV reservoirs by TLR7 agonist in cynomolgus macaques^33^	Combination with immunomodulators (e.g., IL-15)
Permanent reservoir silencing	“Block and lock” (e.g., LEDGINs)	Transcriptional silencing of provirus	Hybrid cure	Long-term safety validation; long-term silencing stability^35^	Transcriptional silencing of HIV-1 by LEDGINs demonstrated in models^35^	Combination with CRISPR-based epigenetic editing for enhanced silencing
Cell replacement/editing	CCR5-edited stem cell transplant	CCR5 disruption & allogeneic immune clearance	Sterilizing cure	Donor matching limitations; high transplantation risks (e.g., GVHD); ethical concerns	Sustained remission via CCR5-Δ32 HSCT (Berlin/London patients);^44^ Remission with wild-type CCR5 HSCT (City of Hope patient)^45^	HIV-1 resistance conferred in CRISPR-edited iPSCs^44^
Cellular Therapy	CAR-T cell therapy	Killing of infected cells by engineered T cells	Functional cure	Viral escape mutations; T-cell exhaustion; limited *in vivo* persistence	Dual CD4-CAR reduces viral load in BLT humanized mice;^40^ M10 CAR-T + chidamide achieved 67.1% mean viral load reduction in Phase I/II trial^37^	Combination with CRISPR editing (e.g., CCR5 knockout);^39^ Engineering to secrete bNAbs

*Abbreviations*: bNAb, broadly neutralizing antibodies; SOSIP, soluble, cleavage-independent, prefusion-optimized; LNP, lipid nanoparticle; CAR-T, chimeric antigen receptor T; ART, antiretroviral therapy; LRA, latency-reversing agent; SIV, simian immunodeficiency virus; iPSC, induced pluripotent stem cell; GVHD, graft-versus-host disease; TLR, toll-like receptor; HSCT, hematopoietic stem cell transplantation; BLT, bone marrow-liver-thymus; CTLA-4, cytotoxic T-lymphocyte-associated protein 4.

## 6 Summary and outlook

In the post-ART era, multimodal synergistic strategies toward HIV-1 cure exhibit considerable promise. This review has systematically summarized key technological advancements and their potential for integration. Specifically, stabilized native-like Env trimers delivered via mRNA-LNP platforms effectively enhance the induction and maturation of bNAbs; CRISPR/Cas9 systems, when combined with long-acting antiretroviral formulations (e.g., LASER ART), have reduced latent reservoirs by over 50% in preclinical models; “shock and kill” strategies utilizing LRAs are being synergized with immune effector mechanisms, including CAR-T cells and bsAbs; and stem cell-based interventions, in conjunction with immune checkpoint modulation, provide innovative pathways for sustained remission. Collectively, these approaches constitute a multi-tiered framework focused on reducing latent HIV-1 reservoirs, reconstituting immunity, and silencing proviruses.

Nevertheless, substantial challenges persist, necessitating targeted optimization for each modality. For antibody-based strategies, future efforts should focus on structure-guided immunogen design for conserved epitopes, the development of novel adjuvants that specifically enhance GC-Tfh cell function, and the optimization of delivery platforms (e.g., mRNA-LNP) to enable sustained antigen expression. For genome editing strategies, optimization should include the development of high-fidelity Cas variants, the engineering of novel delivery vectors with improved tissue tropism toward latent HIV-1 reservoir sites, and the combination with immunotherapies to eliminate residual unedited latent reservoirs. For reservoir-targeting therapies, enhancing “shock” efficacy requires developing synergistic LRA combinations tailored to latent reservoir heterogeneity, while improving “kill” potency necessitates immune priming with cytokines or immune checkpoint inhibitors prior to latency reversal. For cellular therapies, engineering solutions should incorporate HIV-resistance mechanisms (e.g., CCR5 editing), features that counter T-cell exhaustion, and modifications that enhance cellular persistence.

Beyond optimizing individual strategies, future progress in HIV-1 cure research will rely on orchestrated multimodal therapies rather than single-modal interventions. Success will hinge on designing regimens that synergistically combine strategies to offset their individual limitations. For instance, genome editing could disrupt major reservoirs, while antibody-based therapies provide immediate viral control and clear residual HIV-1-infected cells. The “shock and kill” strategy must be augmented by immune priming prior to latency reversal, followed by targeted killing using bsAbs or CAR-T cells. A key challenge lies in defining the optimal sequence, timing, and dosing of these combinatorial regimens.

Looking ahead, the integration of artificial intelligence (AI) is poised to revolutionize the development of multimodal HIV-1 cure strategies. AI-driven models can accelerate immunogen design by predicting the structures of native-like Env trimers and simulating the maturation pathways of bNAbs. In genome editing, AI algorithms can optimize gRNA design to maximize on-target editing efficiency and predict potential off-target effects. AI-powered analysis of multi-omics data could yield predictive insights into latent reservoir dynamics and treatment responses, thereby facilitating the development of personalized cure regimens tailored to individual viral and immune profiles.

Additionally, substantial real-world barriers impede the clinical translatability of these multimodal strategies. The high cost of these advanced therapies poses challenges to their scalability and equitable access, particularly in low-income and middle-income countries with a high HIV-1 burden. Ethical considerations demand careful attention, particularly regarding the risks of germline gene editing and the ethical implications of allogeneic stem cell transplantation. Implementation of these strategies requires sophisticated healthcare infrastructure, cold-chain logistics, and specialized training, which is often unavailable in resource-limited settings. Future efforts must therefore address these pragmatic challenges through innovations to reduce costs, tiered pricing strategies, the development of ethical frameworks, and capacity-building for global health systems.

In conclusion, the future of HIV-1 cure research depends on orchestrated multimodal therapies, which combine immune modulation, genome editing, latent reservoir targeting, and cellular engineering—administered in optimized sequences and dosages. Success will depend on the development of more predictive preclinical models, ones that better recapitulate human HIV-1 immunity, and adaptive clinical trials designed to evaluate these complex therapeutic regimens. Through sustained interdisciplinary collaboration and a commitment to equitable clinical translation, the goal of curing HIV-1 remains achievable. This goal holds the promise of overcoming the limitations of lifelong ART and providing a transformative paradigm for the cure of HIV-1 and other chronic viral infections.

## CRediT authorship contribution statement

**Qiongbin Mao:** Writing – original draft, Investigation, Data curation. **Lin Li:** Writing – review & editing. **Hongling Wen:** Writing – review & editing.

## Informed consent

Not applicable.

## Organ donation

Not applicable.

## Ethical statement

Ethics approval was waived for this work because no patients' data were reported.

## Data availability statement

Data sharing is not applicable to this review article as no databases were generated or analyzed.

## Animal treatment

Not applicable.

## Generative AI

Not applicable.

## Funding

This work was supported by the grant 2022YFC2604000.

## Declaration of competing interest

Professor Hongling Wen is the member of the *Infectious Medicine* Editorial Board. To minimize bias, she was excluded from all editorial decision-making related to the acceptance of this article for publication. The remaining authors declare no conflict of interest.
